# The Mutual Interactions between Mesenchymal Stem Cells and Myoblasts in an Autologous Co-Culture Model

**DOI:** 10.1371/journal.pone.0161693

**Published:** 2016-08-23

**Authors:** Agnieszka Kulesza, Anna Burdzinska, Izabela Szczepanska, Weronika Zarychta-Wisniewska, Beata Pajak, Kamil Bojarczuk, Bartosz Dybowski, Leszek Paczek

**Affiliations:** 1 Department of Immunology, Transplant Medicine and Internal Diseases, Transplantation Institute, Medical University of Warsaw, Warsaw, Poland; 2 Mossakowski Medical Research Centre, Electron Microscopy Platform, Polish Academy of Science, 5 Pawińskiego St., Warsaw, Poland; 3 Department of Immunology, Center of Biostructure Research, Medical University of Warsaw, Warsaw, Poland; 4 Department of Urology, Medical University of Warsaw, Warsaw, Poland; 5 Warsaw University of Life Sciences – SGGW, Department of Physiological Sciences, Faculty of Veterinary Medicine, 159 Nowoursynowska St., Warsaw, Poland; 6 Postgraduate School of Molecular Medicine, Medical University of Warsaw, 61 Żwirki i Wigury St., Warsaw, Poland; 7 Department of Bioinformatics, Institute of Biochemistry and Biophysics, Polish Academy of Sciences, 5a Pawińskiego St., Warsaw, Poland; Faculty of Animal Sciences and Food Engineering, University of São Paulo, BRAZIL

## Abstract

Both myoblasts and mesenchymal stem cells (MSC) take part in the muscle tissue regeneration and have been used as experimental cellular therapy in muscular disorders treatment. It is possible that co-transplantation approach could improve the efficacy of this treatment. However, the relations between those two cell types are not clearly defined. The aim of this study was to determine the reciprocal interactions between myoblasts and MSC *in vitro* in terms of the features important for the muscle regeneration process. Primary caprine muscle-derived cells (MDC) and bone marrow-derived MSC were analysed in autologous settings. We found that MSC contribute to myotubes formation by fusion with MDC when co-cultured directly, but do not acquire myogenic phenotype if exposed to MDC-derived soluble factors only. Experiments with exposure to hydrogen peroxide showed that MSC are significantly more resistant to oxidative stress than MDC, but a direct co-culture with MSC does not diminish the cytotoxic effect of H_2_O_2_ on MDC. Cell migration assay demonstrated that MSC possess significantly greater migration ability than MDC which is further enhanced by MDC-derived soluble factors, whereas the opposite effect was not found. MSC-derived soluble factors significantly enhanced the proliferation of MDC, whereas MDC inhibited the division rate of MSC. To conclude, presented results suggest that myogenic precursors and MSC support each other during muscle regeneration and therefore myoblasts-MSC co-transplantation could be an attractive approach in the treatment of muscular disorders.

## Introduction

Skeletal muscle is a dynamic tissue with high regenerative capacity since it is exposed to recurrent injuries. Satellite cells are the most important and well-described myogenic stem cell population [1]. Those quiescent sublaminar cells differentiate upon activation into myoblasts, which are muscle progenitor cells. Satellite cells are primarily responsible for muscle growth and regeneration throughout life [2]. However, this niche is partially supplemented throughout life by cells from other compartments, especially from bone marrow. These cells are mobilized into blood and directed by the concentration of chemokines and growth factors to skeletal muscles during exercise or injury [3–5], where they contribute to muscle regeneration process. It is believed that mesenchymal stem cell (MSC), not the hematopoietic fraction is predominantly responsible for supporting satellite cells [6]. Both myoblasts and bone marrow-derived mesenchymal stem cells were previously considered to be a material for cell-based therapy in different muscular dysfunctions [7–9]. Myoblasts present high myogenic activity and their contribution to muscle regeneration after intramuscular injection is well documented [10, 11]. The key problem associated with myoblasts transfer therapy is that the vast majority of injected cells are eliminated from the site of delivery within the first few days even after autologous transplantation [12, 13], which limits their support of muscle regeneration. There are several potential causes of poor myoblasts survival after intramuscular administration: one of the proposed reasons of graft elimination is the exposure to oxidative stress in the site of injection [14, 15], which can be associated with innate immune reaction [12]. As opposed to myoblasts, mesenchymal stem cells possess limited potential to differentiate into striated muscle fibers. The induction of MSC to differentiate into skeletal myogenic pathway was proved possible [16], but its efficacy was rather poor [17]. On the other hand, MSC possess well documented high secretory activity and are believed to stimulate progenitor cells by paracrine mechanism [18].

Both populations of cells, myoblasts and MSC, take part in the muscle regeneration, but possess different characteristics. The objective of this study was to evaluate *in vitro* the mutual influence of myoblasts and mesenchymal stem cells on their features important for the muscle regeneration process. Particularly, we aimed to assess and compare the proliferation rate, migration capacity, myogenic differentiation potential and the susceptibility to oxidative stress of myoblasts and MSC cultured together or under the influence of soluble factors from the other population. The study was carried out in order to understand the processes occurring physiologically in muscles *in vivo*, as well as to provide a framework for the procedure of myoblasts-MSC co-transplantation.

## Material and Methods

**Fig 1:**
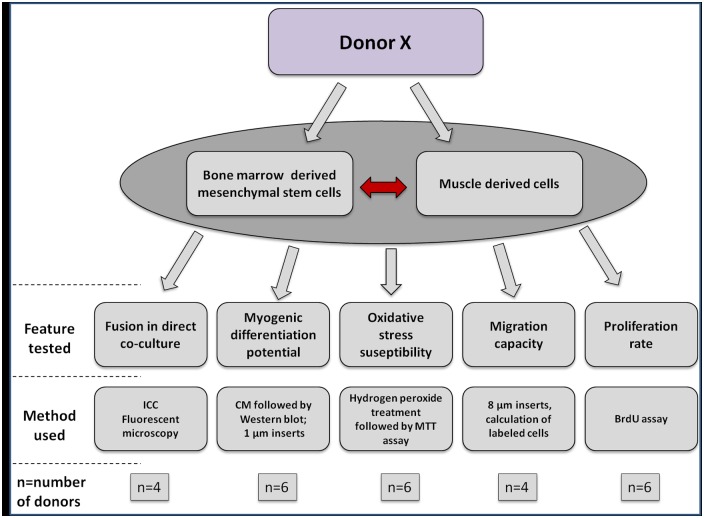
The schematic presentation of experiments and methods used in this study. The interactions were analysed on myoblasts and bone marrow mesenchymal stem cells derived from the same donor.

Bone marrow aspirates and skeletal muscle samples used as the source of cells in this study were collected from 26 adult female Polish white landrace goats. All animals were older than 6 years old. Both MSC and muscle derived cells were isolated from each donor and all analyses were carried out in autologous scheme. The set of experiments which were performed within this study is presented in Fig 1.

This study was carried out in strict accordance with the recommendations in the Guide for the Care and Use of Laboratory Animals of the National Institutes of Health. The origin of animals and the protocol was approved by the Local Ethics Animal Welfare Commission of the Medical University of Warsaw (Permit number: 39/2012). The animals were purchased from goat herd nr PL2000013. During the experiment they were maintained with constant access to food and water in groups of 4–6 animals. They could move freely in pens of appropriate dimensions. The biopsy procedures were performed in anesthetised goats. Animals were sedated with 0.4 mg/kg of midazolam intramuscularly (Midanium^®^, Polfa Warszawa S.A.), followed by intravenous administration of propofol in bolus (2–4 mg/kg b.w. depending on the reaction, Propofol, Scanofol^®^, ScanVet). The anesthesia was maintained with isoflurane (2%, Aerrane, Baxter Polska). Fentanyl was used as analgesic agent. Bone marrow aspirate (about 10 ml) was collected from a radial bone to a probe with 500 U of heparin. A skeletal muscle sample (about 1 g) was taken from *vastus medialis* muscle and placed in a sterile phosphate buffered solution (PBS; Invitrogen) supplemented with 1% of Penicilin-Strepromycin (Invitrogen) solution. After the procedure, the animals were monitored daily by veterinarian to ensure their good condition. If needed, analgesic agents were administrated.

### Isolation and culture of caprine MSC and muscle-derived cells

Bone marrow samples were mechanically disintegrated, diluted with sterile phosphate buffered saline (PBS; Invitrogen) and centrifuged for 5 min at 300*g*. The supernatant was discarded, and the cell pellets were seeded on one 100 mm culture dish (BD Bioscience) and cultured in standard growth medium (GM) consisting of Dulbecco's modified Eagle's medium with low glucose concentration (DMEM-LG, Sigma Aldrich), supplemented with 10% fetal bovine serum (FBS; Life Technologies), 100 U/mL penicillin-streptomycin and 0.5% Fungizone^®^ solution (Life Technologies).

Muscle-derived cells, a population predominantly consisting of myoblasts [12], was isolated from *vastus medialis* muscle. Tissue samples were cleaned from connective tissue, washed in PBS and minced into small pieces. Afterwards, cells were enzymatically dissociated with 0.15% (wt/vol) protease (Sigma, P8811) in DMEM at 37°C with continuous stirring. After 60 min of incubation, the enzyme was inactivated by adding 10% FBS. Cell suspensions were filtered through a 70 μm cell strainer. Cells were washed twice in PBS, re-suspended in growth medium for muscle-derived cells (MDC) consisting of GM supplemented with 5% horse serum and seeded on a 100 mm culture dish (BD Primaria^™^). Preplating procedures were applied after 1 h of incubation. Cells were cultured under standard conditions (37°C, 5% CO_2_, 95% humidity). Fresh growth medium was replaced every two-three days. When 80% confluence was reached, cells were passaged by harvesting with a 0.25% trypsin and 0.02% EDTA solution (Life Technologies). After the 2nd or the 3rd passage, the cells were cryopreserved for further analyses. All assays were performed on cells between the 3rd and the 5th passage.

### Differentiation potential of the isolated populations

To prove the multipotent properties, MSC were cultured with specific media supplements inducing differentiation into adipogenic, chondrogenic and osteogenic lineages. (Human Mesenchymal Stem Cell Functional Identification Kit; R&D Biosystems). After three weeks of differentiation, cells were fixed in 4% (w/v) paraformaldehyde and stained with Oil Red O (Sigma-Aldrich), Alizarin Red solution (Sigma-Aldrich) or Safranin O, respectively.

For MDC, the ability to differentiate into myotubes was assessed. Briefly, cells were cultured in myogenic differentiating medium (mDM) composed of DMEM with 4% HS and 10 μg/mL of insulin. Additionally, immunocytochemical stainings against desmin and sarcomeric myosin were performed. For this procedure, fixed cells were permeabilized with ice-cold methanol for 20 min. The non-specific binding of antibodies was blocked by 30 min incubation in a solution of 1% bovine serum albumin and 5% normal donkey serum in PBS. Primary monoclonal mouse anti-desmin antibody (clone D33, Dako, 1:30) or monoclonal mouse anti-sarcomeric myosin (clone MF20, Developmental Studies Hybridoma Bank, 1:30) were used (overnight, 4°C). After washing, the cells were incubated with donkey anti-mouse secondary antibody conjugated with Alexa-Fluor^®^ 594 (Jackson ImmunoResearch Europe, 1:100, 60 min, RT).

### Cell labeling

For the assessment of interactions in a direct co-culture, MSC were transfected with pMAX-GFP plasmid (Amaxa Biosystems) using the Nucleofector system (Amaxa Biosystems). Typically, 2x10^6^ cells were in 100 μl Cell Line Nucleofector Human MSC Solution and mixed with 2 μg plasmid DNA. Nucleofections were performed using the Amaxa Nucleofector II device with a U-023 program. Post-nucleofection cells were incubated overnight in a RPMI medium supplemented with 20% FBS and used for the following procedures. In one experiment, muscle-derived cells were labeled with red membrane fluorochrome PKH26, and MSC were labeled with green PKH67 (both from Sigma Aldrich). Staining was performed according to the manufacturer instructions with a 4 nM of PKH solution for 4 min in RT. The labeling efficiency was evaluated with fluorescence microscopy (Olympus IX51) and is demonstrated in the supplementary material (S1 Fig).

### Fusion assessment—direct MDC and MSC co-culture

Two different experiments with a direct co-culture of MSC and MDC were conducted. First, PKH67-labeled MSC and PKH26-labeled MDC were co-seeded on a 100 mm culture dish at a 1:1 ratio. After 24 h of incubation, the cells were fixed with a 4% (w/v) paraformaldehyde solution and the nuclei were visualized with DAPI (20 ng/mL, 6 min). The effects were observed and documented with fluorescent microscope Olympus IX51. Short term experiment was performed to avoid artifacts caused by diffusion of membrane dyes from one cell type to another. In the second experiment, GFP-labeled MSC were seeded onto unlabeled MDC culture (2^nd^ day of culture) and incubated for 24 or 48 h with myogenic medium containing 4% HS and insulin. Then, a co-culture was fixed with 4% PFA, immunostained with anti-desmin antibody as described above, and cell nuclei were visualized with DAPI.

### The effect of MSC-soluble factors on myogenic MDC differentiation

Indirect co-culture was conducted to evaluate the effect of MSC-derived soluble factors on MDC myogenic differentiation. For this experiment, MDC were seeded on a 12-well plate in a concentration of 5x10^4^/well. At the same time, 2x10^4^ MSC or MDC (as a control) were seeded on inserts with 1 μm pore size (Thincert^™^, Greiner) which allow only for diffusion of soluble factors and not for the cells migration. Cells were left to attach and after 24 h the inserts were transferred to the wells with MDC seeded in the lower compartment. After 3 days of culture in a myogenic differentiation medium, the cells in the basal compartment were fixed and stained with DAPI as described above. The fusion index (FI) was calculated as a ratio of nuclei within the myotubes (>2 nuclei) to the total number of nuclei in at least 10 randomly chosen fields of view per well. The duration of experiment was determined on the basis of preliminary data—in longer term culture in mDM caprine derived myotubes started to detach from the culture flask what would affect the FI calculations. For a co-culture, MDC and MSC were always used from the same animal. Cells from 6 donors were analysed.

### The effect of MDC-conditioned medium on myogenic MSC differentiation

To evaluate the effect of MDC-derived soluble factors on MSC myogenic differentiation, mesenchymal stem cells were cultured in a MDC-conditioned medium in autologous manner (paired populations derived from the same donor). Conditioned medium (CM) was collected every second day from a fully confluent MDC cultured in mDM. The CM was pushed through a 0.22 μm filter and mixed with standard GM in 1:1 ratio. As control, MDC were cultured in a standard GM or GM with fresh mDM in 1:1 ratio. After 6 days, the cells were collected and cell pelletes were stored at -80°C for Western blot analysis.

### Western blotting

Cellular homogenates were prepared using RIPA lysis buffer supplemented with protease inhibitors: phenylmethylsulfonyl fluoride (0.4 mM), aprotinin (10 μg/ml), and sodium orthovanadate (10 μg/ml) (Sigma Aldrich). For western blot analyses, equal amount of total cellular proteins were first dissolved by 12% SDS—PAGE electrophoresis, and next transferred at 125 mA for 2 h to PVDF membranes. After blocking non-specific binding with 5% non-fat dry milk (RT, 1 h), the membranes were incubated overnight at 4°C with mouse monoclonal anti-desmin or rabbit polyclonal anti-GAPDH antibodies (1:200, clone D33, Dako and 0.2 μg/ml, G9545, Sigma Aldrich, respectively), followed by 1 h (RT) of incubation with the secondary donkey anti-rabbit or anti-mouse antibodies conjugated with HRP (1:1000, Cell Signaling Technology). The blots were developed using chemiluminescence western blotting detection reagent (Amersham International).

### Viability of MDC, MSC and the co-culture after H_2_O_2_ treatment

To compare the susceptibility to oxidative stress *in vitro*, MDC, MSC and MDC-MSC mixture (1:1) were seeded on a 96–well flat-bottomed microplate. Once full confluence was reached, cells were exposed to GM with various concentrations of H_2_O_2_: 500, 600, 700, 800, 900, 1000, 1200 μM (100 μl per well). The assessment of cell viability was based on the ability to reduce soluble 3-(4,5-dimethylthiazol-2-yl)-2-5-diphenyltetrazolium bromide—MTT (Sigma Aldrich)–into an insoluble purple formazan reaction product. After 22 h of incubation, a 20 μl of MTT solution (5 mg/ml in PBS) was added to each well for 2 h. Then, MTT solution was removed, and formazan was dissolved by immediate addition of 100 μL dimethyl sulfoxide (Sigma Aldrich). The absorbance of the sample in each well was measured at 540 nm using a microplate reader (BIOTEK Power Wave XS) with a reference wavelength of 650 nm. The experiments were performed in triplicates on cells derived from 6 different animals. For a co-culture, MDC and MSC were always used from the same animal.

### Migration assay

The migration ability of analysed populations was studied using cell culture inserts with 8 μm pore size (Thincert^™^, Greiner). Approximately 1.5x10^4^ MDC or MSC labeled with green fluorochrome PKH67 (Sigma Aldrich) were seeded on inserts. In the basal compartment, there was either no cells (unstimulated migration) or unlabeled MDC or MSC seeded in a concentration of 1.5x10^4^/well on 24-well bottom plates (SensoPlate, grainer). The cells were incubated at 37°C and 5% CO_2_ for the next 72 h to allow cell migration from the inserts to the basal compartment. Afterwards, inserts were removed, cells from the wells were fixed with 70% methanol and the nuclei were visualized with DAPI staining (20 ng/mL of DAPI solution for 4 minutes, RT). Results were analysed with a cell imaging multi-mode microplate reader Cytation™ 3 (BioTek), which allowed to specify the number of objects giving a signal of blue (DAPI, wavelength: 377–477 nm) and green fluorescence (PKH 67, wavelength: 469–525 nm). The number of migrating/stimulating cells was calculated from 20 different fields of view in each sample. Fields of view had the same location in the wells for all samples and were chosen arbitrarily before analysis. Paired stimulating and stimulated populations were always derived from the same donor, n = 4.

### Proliferation assay

The rate of cell proliferation was determined using a colorimetric 5-bromo-2'-deoxyuridine (BrdU) proliferation ELISA immunoassay (Roche). MDC and MSC were seeded into 96-well plates at a density of 8x10^3^ cells per well and cultured in 4 different culture media: 1) standard GM, 2) GM supplemented with bFGF (10 ng/ml), 3) MDC conditioned medium, and 4) MDC conditioned medium. For the experiment, MDC and MSC were used from the same donors (n = 6). The conditioned media were collected from confluent cultures after 24 h, filtered through 0.22 μm pores and used without additional dilution. After 36 h, BrdU was added and during the next 12 h of incubation it was incorporated into the DNA of proliferating cells. Furtherly, immunostaining was performed according to the manufacturer instructions. The absorbance was measured at wavelength 450 nm (BioTek PowerWave XS). The experiment was performed in triplicates.

### Statistical analysis

Statistical analysis was performed using Statistica software, version 12 (StatSoft Inc., Tulsa, OK, USA). Quantitative data are presented as a median with the lower and upper quartiles and the range or as the mean ± standard error or standard deviation. Results of the migration assay experiments were analysed using non-parametric tests: either U-Mann Whitney test or Kruskal-Wallis test, depending if two or more groups were compared, respectively. Data from proliferation assays were analysed with Wilcoxon signed-rank test for related pairs. Results of the MTT assay were analysed with Wilcoxon signed-rank test in comparison to control values or with Kruskal-Wallis test when ratios of sample absorbance/internal control absorbance between three different groups were compared. *P*-values lower than 0.05 were considered be statistically significant.

## Results

Before performing specific experiments which aimed at evaluation of interactions between MDC and MSC, the identity of the isolated populations was confirmed. Both populations displayed fibroblastic, spindle shape morphology, were clonogenic if seeded in low density and proliferated on plastic surfaces. Muscle-derived cells expressed desmin and were able to differentiate into multi-nucleated myotubes which expressed both desmin and sarcomeric myosin (supplementary material, S2 Fig), whereas MSC possessed standard multilineage differentiation capacity what was confirmed by successful induction of chondrogenic, osteogenic and adipogenic differentiation (S3 Fig).

### MSC get into direct contact when co-cultured both with undifferentiated and differentiating MDC

**Fig 2:**
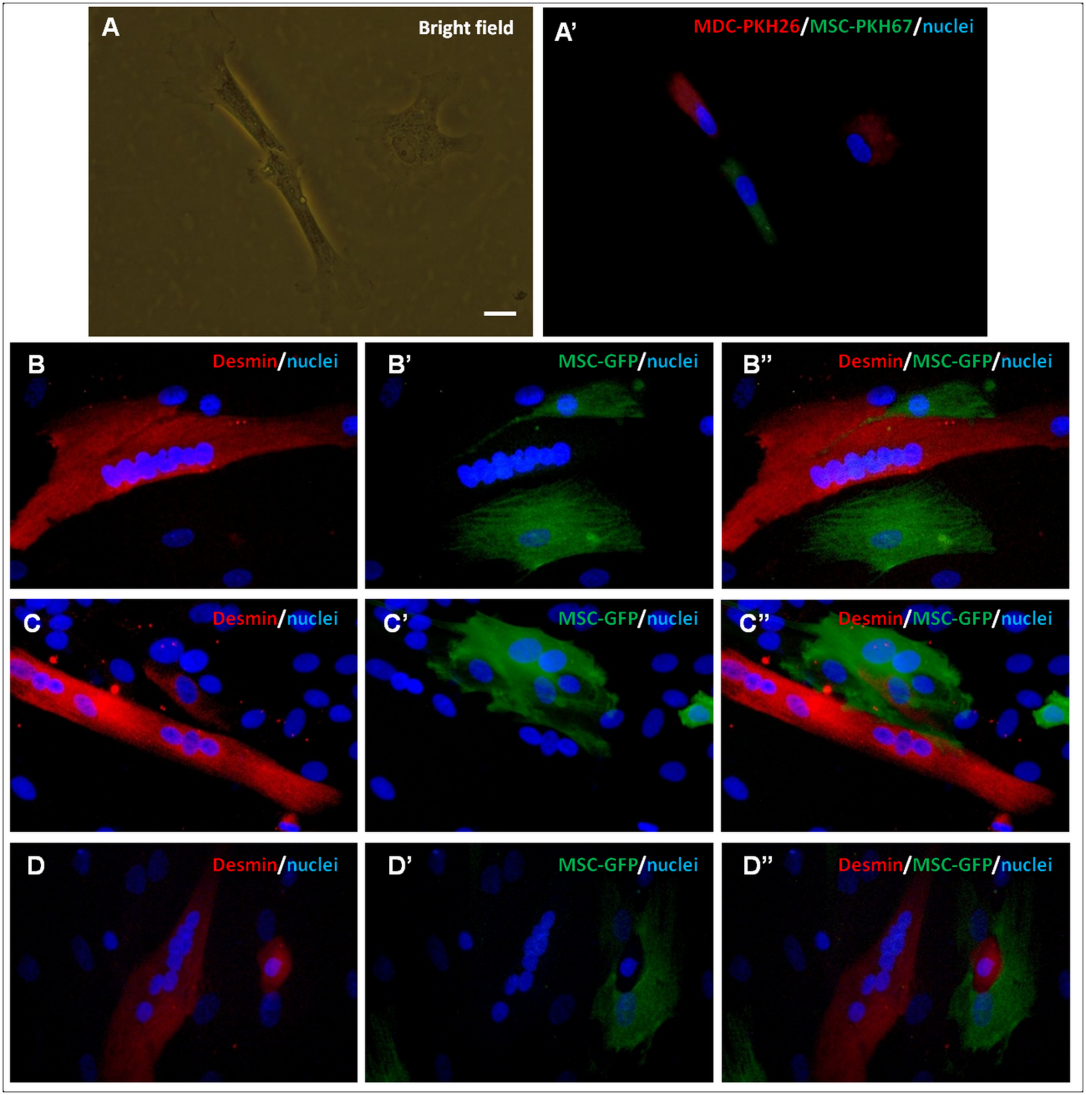
MDC and MSC 24 h in a direct co-culture. Images in rows represent the same fields of view. A. Bright field microscopy. A' Fluorescent microscopy where MDC are red (PKH26) and MSC are green (PKH67), nuclei stained with DAPI. It can be observed that cells tend to contact directly. B,C,D—Fluorescent microscopy, where MSC are green (GFP), desmin is stained with red fluorochrom (AlexaFluor^®^ 594) and nuclei are stained with DAPI (blue). Different types of direct contact can be observed—extending filopodia (B), parallel arrangement of different cell types (C) or surrounding one cell by another (D). Scale bars: 20μm.

To study interactions between two different cell types, first we co-seeded PKH67-labeled MSC with undifferentiated, PKH26-labeled MDC. Already after 1 day the culture showed intercellular contacts and cellular interactions between MDC and MSC could be observed. Moreover, the interactions seemed not to be accidental—cells tended to contact directly even when seeded in relatively low concentration (Fig 2A). Direct contact also took place if GFP(+)MSC were seeded onto attached unlabeled MDC and exposed to the myogenic differentiating medium. After 24 h in co-culture, MSC extended filopodia in direction to differentiating muscle cells (Fig 2B), arranged in parallel to myotubes (Fig 2C) or surrounded the MDC (Fig 2D).

### MSC contribute to muscle structure formation when directly co-cultured with differentiating myoblasts

**Fig 3:**
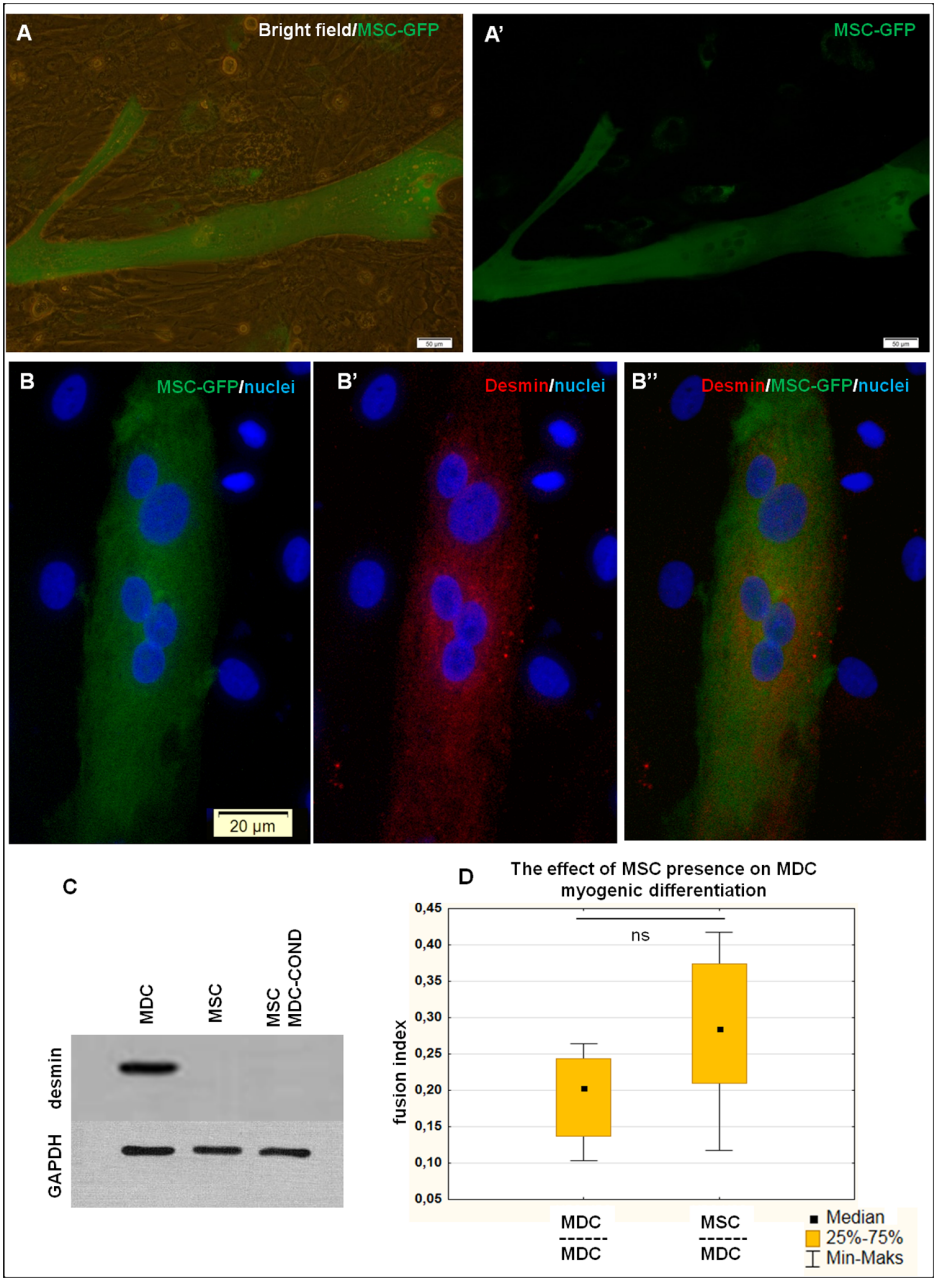
Mutual MDC-MSC interaction on myogenic differentiation. (A, B) Fluorescent microscopy where MDC (unlabeled) and MSC (GFP+, labeling efficiency less than 50%) are in a direct co-culture under myogenic differentiation medium (mDM) for 48 h. Images in rows represent the same fields of view. GFP+ myotubes can be observed what proves the ability of MSC to contribute in the myotube formation. Staining against desmin (B) confirms myogenic character of formed structure. scale bars: (A) 50μm, (B) 20 μm. C. Representative Western blot for desmin expression in MDC, MSC and MSC cultured in MDC-conditioned (COND) medium (50%) (all from one donor). D. Graph presents fusion index of MDC after 3 days of culture in mDM under the influence of soluble factors derived from either MDC or MSC (1 μm inserts).

When co-culture was analysed 48 h after MSC seeding, numerous GFP positive multi-nucleated myotubes could be observed (Fig 3A). Clearly, MSC were able to fuse with differentiating myoblasts and in this way they contributed to the formation of muscle structures. This phenomenon was confirmed by anti-desmin staining—GFP positive multi-nucleated structures displayed expression of this myogenic marker (Fig 3B). No desmin expression was observed in mononuclear GFP+ cells.

### MSC do not acquire myogenic phenotype if exposed to MDC-derived soluble factors

We have used MDC conditioned medium to evaluate, if MDC-secreted soluble factors are able to induce myogenic differentiation of caprine primary MSC derived from the same donor. No myotube formation was observed in any of CM-treated MSC populations (cells from 6 different animals). Additionally, no desmin expression could be detected in MSC after 6 days of treatment (Fig 3C).

### MSC-derived soluble factors do not affect myoblasts differentiation

To evaluate the effect of MSC-derived soluble factors on MDC myogenesis, indirect co-culture assay was used, where MSC or MDC (as control) were placed on inserts Ø 1 μm. After three days of myogenic differentiation the fusion index of MDC placed in the lower compartment was analysed. The median FI rate in the MDC influenced by MSC amounted 28.5%, whereas FI of control MDC achieved 20.3%. The difference was not statistically significant (Fig 3D).

### MSC are more resistant to oxidative stress than MDC, but direct co-culture with MSC does not diminish the cytotoxic effect of hydrogen peroxide on MDC

**Fig 4:**
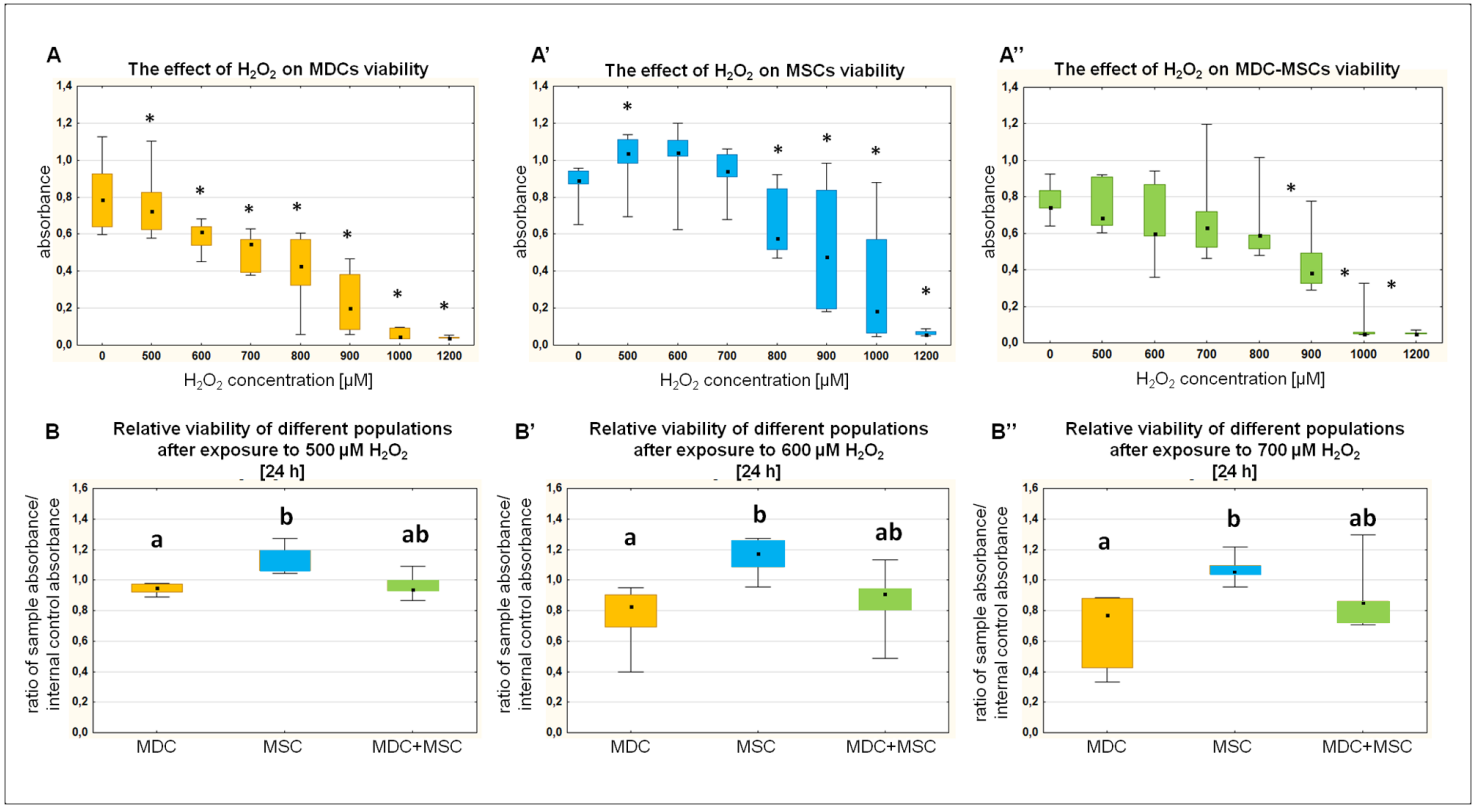
The effect of the *in vitro* oxidative stress on MDC, MSC and MDC-MSC co-culture. Metabolic activity was measured by MTT assay. Data presented as median, interquartile range and min, max. Upper graphs present absorbance values measured in MDC, MSC or MDC-MSC (A,A',A'', respectively) treated with increasing concentrations of H_2_O_2_ analysed by Wilcoxon signed-rank test in comparison to the control group. *, p<0.05; Lower graphs present relative values, which is the ratio of sample absorbance to the control absorbance from the same donor (internal control) obtained after treatment with 500, 600 or 700 μM of H_2_O_2_ (B,B',B'', respectively) analysed with ANOVA Kruskal-Wallis test. Values that differed significantly (p<0.05) are indicated with different lowercase letters. Experiments were done on cells from 6 different donors (same donors for MDC and MSC) and performed in triplicates.

The exposure of both populations derived from the same donors with increasing concentrations of hydrogen peroxide revealed higher susceptibility of MDC to oxidative stress in comparison to corresponding MSC populations. The statistically significant decrease of MDC viability was noted already after exposure to 500 μM H_2_O_2_ (Fig 4A). In the case of MSC, low dose H_2_O_2_ (500 μM) caused significant increase in cell viability in comparison to untreated cells. The significantly cytotoxic effect was observed when 800 μM or higher concentrations of H_2_O_2_ were used (Fig 4A'). To verify the difference between two analysed cell types, data were expressed as a ratio of sample absorbance to the internal control absorbance (untreated cells from the same population) and compared between different cell types. The analysis confirmed that MSC are more resistant to oxidative stress than myoblasts—significant differences were observed at three different concentrations: 500, 600 and 700 μM H_2_O_2_ (Fig 4B, 4B' and 4B'' respectively). Additionally, MDC and MSC derived from the same donors were seeded in a direct co-culture and exposed to oxidative stress at the same scheme as monocultures. The use of 900 μM H_2_O_2_ was necessary to induce significant reduction in metabolic activity of co-cultured population (Fig 4A''). Lower concentrations of hydrogen peroxide did not elicit relevant effect on MDC-MSC viability. To evaluate differences between mono-cultures and co-culture, absorbance values normalised with internal control were compared. The analysis revealed that the resistance to oxidative stress was not increased in the co-culture in comparison to either MDC or MSC in any of the tested H_2_O_2_ concentrations (Fig 4B, 4B' and 4B'').

### MSC possess greater migration ability than MDCs, which is further enhanced by MDC-derived soluble factors

**Fig 5:**
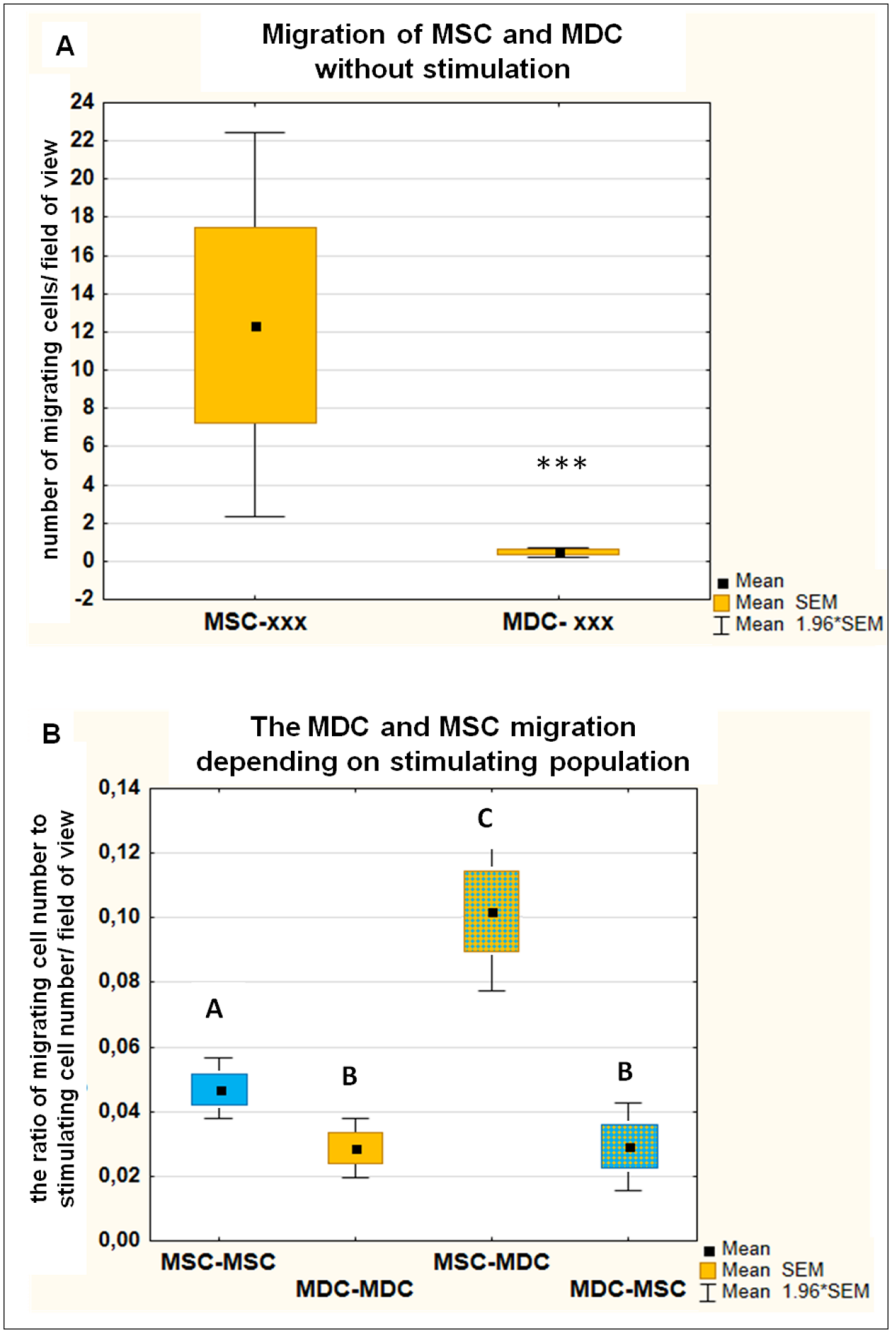
MDC and MSC migration capacity. The migration of MDC and MSC analysed by inserts assay (Ø 8μm). Data presented as mean and SEM. A) The migration rate of unstimulated MDC and MSC (no cells in the lower compartment marked as xxx) analysed with U-Mann Whitney test, ***, p<0.01. B) The graph presents the migration stimulated by cells seeded in the lower compartment. The rate of MDC or MSC migration in relation to the stimulating cell number was calculated. Descriptions on X axis reflect the location of cell types—i.e. MDC-MSC means the MDC cells migrated under stimulation of MSC cells. Data analysed with ANOVA Kruskal-Wallis test. Values that differed significantly (p<0.05) are indicated with different uppercase letters.

We compared migration capacity of MSC and MDC isolated from the same animals using assay with 8 μm Ø inserts. Performed experiments demonstrated that MSC in the standard culture conditions possess significantly higher migration ability than myoblasts (25 fold difference, p = 0.008, Fig 5A). The presence of cells in the lower compartment remarkably increased the migration of both populations if compared to the unstimulated control (p<0.001 for both MSC and MDC, regardless of the stimulating population). The influence of cells seeded in the lower compartment was so strong that we analysed the results in relation to the number of stimulating cells in the field of view. Thus, the ratio of migrating cell number (labeled with green fluorochrome) to the number of stimulating cells (unlabeled) was calculated and compared. After stimulation by the same type of cells (MSC:MSC vs MDC:MDC), MSC possessed significantly higher migration ability than their MDC counterparts (p = 0.002, Fig 5B). Interestingly, MSC exposed to MDC-derived environment migrated with significantly higher intensity than those exposed to MSC (p = 0.018, Fig 4B). Similar effect in regard to MDC population was not observed—the type of stimulating population had no significant influence on MDC migration rate (Fig 5B).

### MSC-derived soluble factors enhance proliferation rate of MDC, whereas MDC have an opposite effect on MSC

**Fig 6:**
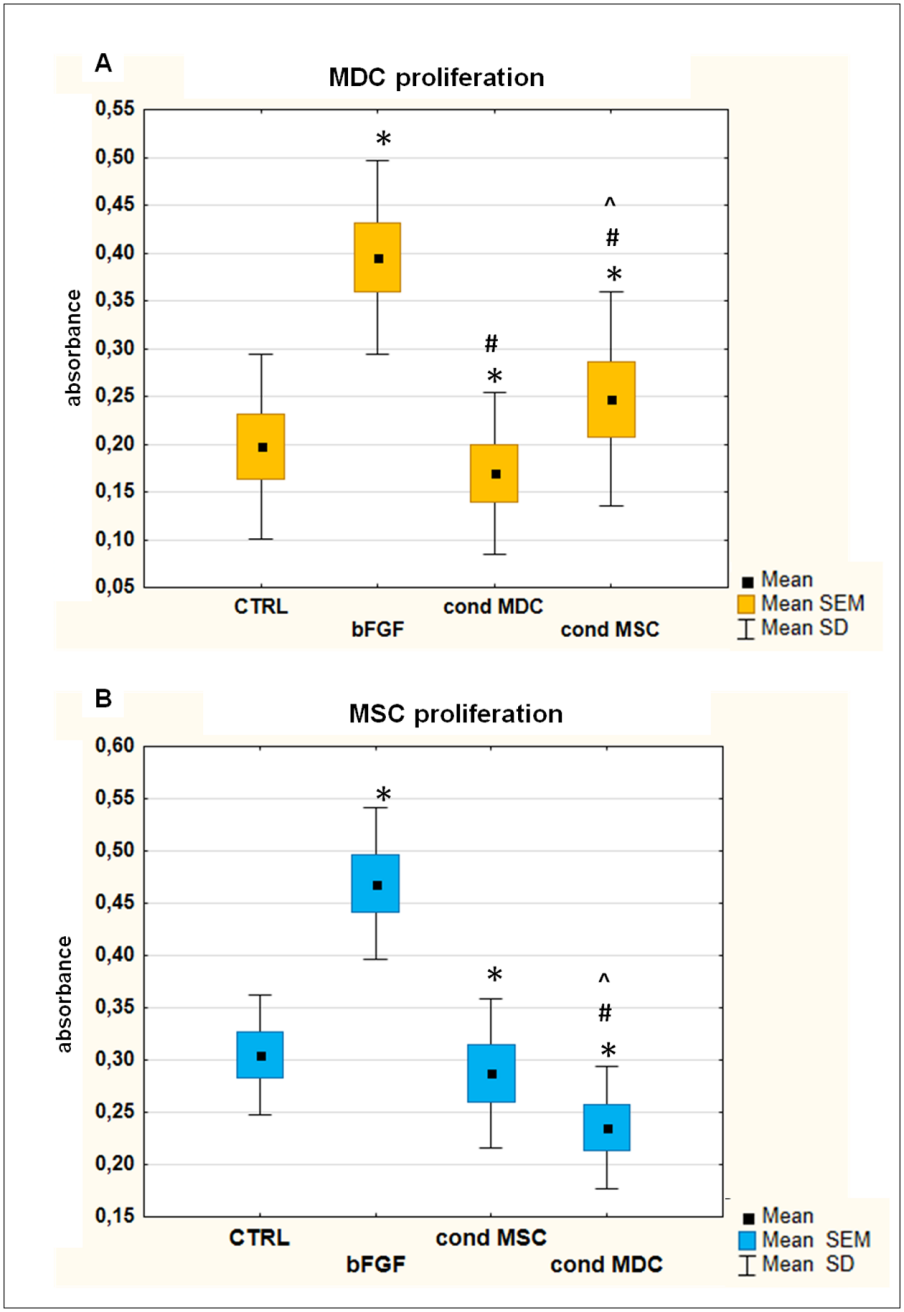
MDC and MSC proliferation rate. Proliferation of MDC (A) and MSC (B) derived from the same donors (n = 6) was measured with BrdU assay. Data presented as means, SEM, SD. The graphs present proliferation of cells cultured in standard GM (CTRL), GM supplemented with bFGF and cells cultured in 100% conditioned medium (cond) derived either from MDC or MSC. Data analysed with Wilcoxon test for related groups. Values that differed significantly (p<0.05) were indicated as following: * in comparison to CTRL group; # in comparison to bFGF group; ^ in comparison to the second conditioned group. Tests were performed in triplicates.

To establish the effect of soluble factors on proliferation rate of both analysed cell types, the cells were cultured in conditioned media (CM) derived from either MDC or MSC (populations derived from the same donors). Basic fibroblasts growth factor (bFGF), a well known mitogen, were used as positive control factor in these experiments. The median proliferation rate of both populations was significantly higher (p<0.05) in response to bFGF in comparison to the untreated control (Fig 6). However, the reaction to bFGF was significantly higher in MDC in comparison to MSC (the increase of median value was 2.09- and 1.43-fold, respectively). The analysis after exposure to conditioned media revealed that MDC-CM significantly decreased proliferation of MDCs when compared to cells cultured in fresh GM. At the same time, MSC-CM significantly enhanced proliferation of MDC in comparison to control MDC, as well as to MDC-CM treated with MDC (Fig 6A). In contrary, proliferation of MSC was inhibited by CM, regardless of cell type used for conditioning. However, MDC-CM arrested MSC to a significantly higher extend than MSC-CM (Fig 6B).

## Discussion

Myogenesis is a complex, multistep process in which several different cell types are involved. Recognition of interactions between these cell types improves our knowledge about naturally occurring muscle regeneration process and potentially updates protocols for muscle-directed cell therapy. Previously, it was demonstrated that co-transplantation of myoblasts and MSC can improve the clinical effect of intramyocardial cell transfer [19–21]. However, the exact mechanisms underlying this synergy were not clearly defined. The aim of this study was to evaluate how these cell types interact with each other. The experiments were performed on caprine cells as it was a part of a larger project performed on goats, in which the effect of intrasphincteric MDC-MSC co-transplantation has been under investigation. The feasibility of isolation and culture of both caprine myoblasts and bone marrow-derived mesenchymal stem cells were previously demonstrated [22, 23]. However, as goats are rarely used as experimental animals, the identity of isolated populations had to be confirmed first. Muscle-derived cells expressed myogenic marker desmin and were able to fuse into myotubes under myogenic conditions. The median fusion index at day 3 amounted 20.3% what is similar to data presented on C2C12 mice cell line, in which fusion index at day 4 was reported to be 20% [24]. Therefore, it can be concluded that isolated population predominantly consisted of myoblasts. However, as heterogeneity of the population isolated from skeletal muscle tissue is well known [25], we’d rather call the obtained population "muscle-derived cells". Bone marrow-derived cells, typically for a MSC population, displayed multilineage differentiation capacity. In the present study, several different features were analysed. First, the effect of direct or indirect presence of myoblasts on MSC myogenic differentiation was studied. We demonstrated that MSC are able to fuse with myoblasts derived from the same donor and both cell types contribute to creation of multi-nuclear myotubes. It is in concordance with previously published data, where hybrid myotubes of human MSC and C2C12 mice myoblasts cell lines were shown [6, 26]. However, the occurrence of fusion does not prove the differentiation capacity. Therefore, we evaluated desmin expression in MSC under influence of myoblasts-derived soluble factors (50% conditioned medium). Our results indicated no unequivocal myogenic differentiation (Fig 3). Mesenchymal stem cells are believed to posses ability to differentiate into skeletal muscle. Nonetheless, results reported by Stern-Streater *et al*. suggest that MSC myogenic potential depends on the cell source. Authors have proved desmin protein expression in MSC derived from adipose tissue treated with myoblasts-conditioned medium, but not in bone marrow MSC [27]. Similarly, Belema-Bedada *et al*. concluded from their *in vitro* experiments that the bone marrow MSC are not able to undergo complete and functional myogenic differentiation [28]. On the other hand, it was shown that *in vivo*, one month after transplantation, bone marrow-derived cells can acquire sublaminar position in muscle fibers and express satellite cells markers which proves their ability to undergo complete differentiation into muscle stem cell [4]. *In vitro*, the entire myogenic differentiation of MSC was achieved only by using multifactoral protocol which included genetic manipulation [29]. Therefore, we can reach the conclusion that MSC can easily fuse with myoblasts and in this way contribute to muscle regeneration process, but to become truly myogenic cells they need either more time than it is tested in *in vitro* protocols (maximally 21 days [27]) or more complex combination of factors and possibly interaction with other cell types which are present in regenerating muscle, not only with myoblasts.

One of the crucial aspects of muscle regeneration is an inflammatory process. Each muscle injury is associated with rapid infiltration of neutrophils (culmination in 6–24 h post-injury), followed by pro-inflammatory M1 macrophages which are subsequently replaced by M2 population. It was shown that innate immune response with particular role of the macrophage phenotype transition is an essential component of muscle regeneration [30]. On the other hand, the phagocytic activity of myeloid cells is associated with locally increased concentration of free radicals. Reactive oxygen species are cytotoxic products of both neuthophils and M1 macrophages. It was previously suggested that oxidative stress can be one of the significant factors responsible for poor myoblast transfer therapy efficacy [14]. The results presented herein demonstrate that MSC are significantly less susceptible to oxidative stress than myoblasts. Previously, the superior resistance to oxidative stress in comparison to myoblasts was demonstrated in muscle-derived stem cells [31]. Moreover, it was postulated that increased antioxidant capacity can be a critical property for survival after intramuscular transplantation [15]. Thus, it is possible that mesenchymal stem cells after transplantation could display higher survival rate than myoblasts. Interestingly, our results suggest that the MDC-MSC co-transplantation approach would not save MDC from cytotoxic effect of oxidative stress as a direct MSC-MDC co-culture was not significantly more resistant to hydrogen peroxide than MDCs alone. Possibly, the increased resistance to oxidative stress demonstrated in MSC is associated with intrinsic antioxidant capacity which is not transferable to adjacent cells.

Another important feature, both in physiological processes and in cell therapy, is cell mobility. Our results demonstrated that unstimulated MSC displayed about 25-fold higher ability to migrate than myoblasts (p = 0.008, Fig 5A). The poor migration capacity of myoblasts *in vivo* was reported by others [32, 33] and has been indicated as one of the features responsible for myoblasts transfer therapy failure [34]. The ability to migrate effectively from the site of delivery potentially increases the area which can be influenced by transplanted cells. Attempts made to improve the migration of transplanted myoblasts included usage of different growth factors (i.e. Fibroblast Growth Factor, Insulin Growth Factor-1, Nerve Growth Factor or Vascular Endothelial Growth Factor) or metalloproteinases [33, 35]. As majority of those factors are secreted by mesenchymal stem cells, what was demonstrated in human [36] or mice [37] MSC, we hypothesized that the presence of MSC could enhance myoblasts migration. Our results demonstrated that indeed the MSC presence significantly increased MDC migration rate (p<0.0001), but not to a higher extent than MDC did. It can be concluded that either key factors which stimulated MDC migration were secreted by both cell types at similar levels or that there were other contradictory stimuli secreted by MSC that diminished pro-migratory effect. One of the possible mechanisms could be simultaneous enhancement of MDC differentiation. It is known that once the fusion process is initiated, the migration capacity is reduced [38]. Therefore, we tested in a separate experiment if the presence of MSC affects the MDC fusion. The median fusion index value of MDC exposed to MSC paracrine effect increased by 40% in comparison to the MDC-exposed cells, the difference, however, did not reach statistical significance.

Similarly to MDC, MSC migration rate was significantly increased if stimulation by any cells seeded in the well bottom below inserts took place (p<0.0001 in comparison to unstimulated cells, regardless of the stimulating population). Moreover, the migration capacity of stimulated MSC was greater than stimulated MDC (regardless if they were stimulated by the same or by the second population). Such finding was expected as MSC express wide variety of chemokines receptors and are able to respond actively to chemotactic stimuli [39]. Interestingly, MSC displayed significantly higher migration rate when triggered by MDC than by MSC (p = 0.018, both stimulating populations always derived from the same donor). Myoblasts are normally present in a regenerating muscle tissue as they are the progeny of activated satellite cells. Thus, it seems reasonable from a physiologic point of view that factors released by this population increase recruitment of circulating MSC to the damaged site. It might be that tumor necrosis factor alpha (TNF-α) is involved in this process. It was previously demonstrated that this pro-inflammatory cytokine enhances susceptibility of MSC to chemotactic factors [40]. On the other hand, it well known that TNF-α is highly expressed in injured muscle and that it is secreted not only by the inflammatory cells, but also by myoblasts and myotubes [41].

We also tested the reciprocal interactions between analysed populations in terms of their proliferation rate. It was shown that the survival of myoblasts in the transplantation site is poor even after an autologous transfer [12, 13]. The improvement of survivor cells proliferation is one of the possible approaches to enhance cell transfer efficacy. MSC are known to secrete many growth factors, thus, we hypothesized that MSC conditioned medium would increase myoblasts proliferation. Our results confirmed this assumption, as proliferation rate of MDC cultured in 100% MSC-CM was 25% higher than those cultured in standard growth medium (p = 0.02). Similar effect of bone marrow MSC on mice C2C12 cells was previously reported [9]. The authors presented evidence that myoblastic response was mainly dependent on the paracrine release of vascular endothelial growth factor (VEGF) by MSC. However, MSC secrete also IGF-1, HGF or bFGF [42], which are known to stimulate myoblasts [43], and this can be potentially additional way of MSC paracrine action.

In conclusion, based on results presented herein, the following set of interactions can be proposed: the myoblasts enhance migration of MSC which can increase their recruitment from blood stream to the site of muscle injury. In turn, MSC increase myoblasts proliferation and in this way can improve muscle regeneration. Proliferating myoblasts inhibit divisions of MSC which can be a signal to switch into differentiation pathway. Finally, MSC are more resistant to oxidative stress, so possibly serve as a regenerative pool in more serious injuries associated with accelerated concentration of reactive oxygen species. Additionally, interactions demonstrated between myoblasts and mesenchymal stem cells suggest that co-transplantation of these two cell types can improve cell therapy efficacy in cases of muscular disorders.

## Supporting Information

S1 Fig Cell labeling efficiency. A, A', A'' represent the same field of view—caprine MSC two days after nucleofection with GFP encoding gene. Less than 50% of cells display distinct green fluorescence. B) Caprine MDC 2 days after labeling with PKH26 (100% efficiency); C) Caprine MSC 2 days after labeling with PKH67 (100% efficiency). Scale bars: A—200 μm, B,C—50 μm.
(TIF)

S2 Fig Myogenic identity of caprine MDC. Images in rows represent the same field of view: A) Undifferentiated MDC; B, C) MDC differentiated into myotubes. A, B) Desmin is stained in red (Alexa Fluor^®^ 594), nuclei are stained in blue (DAPI); C) Sarcomeric myosin is stained in red (Alexa Fluor^®^ 594), nuclei are stained in blue (DAPI). Scale bars: A—50 μm, B,C—200 μm, C—20 μm.
(TIF)

S3 Fig Differentiation potential of caprine MSC. A) Undifferentiated MSC; MSC differentiated into adipocytes (B), osteocytes (C) and chondrocytes (D).
(TIF)
