# Supplementary figures and images for: The Mutual Interactions between Mesenchymal Stem Cells and Myoblasts in an Autologous Co-Culture Model
Source: PLoS One. 2016 Aug 23;11(8):e0161693. doi: 10.1371/journal.pone.0161693 (PMC4994951; doi:10.1371/journal.pone.0161693)

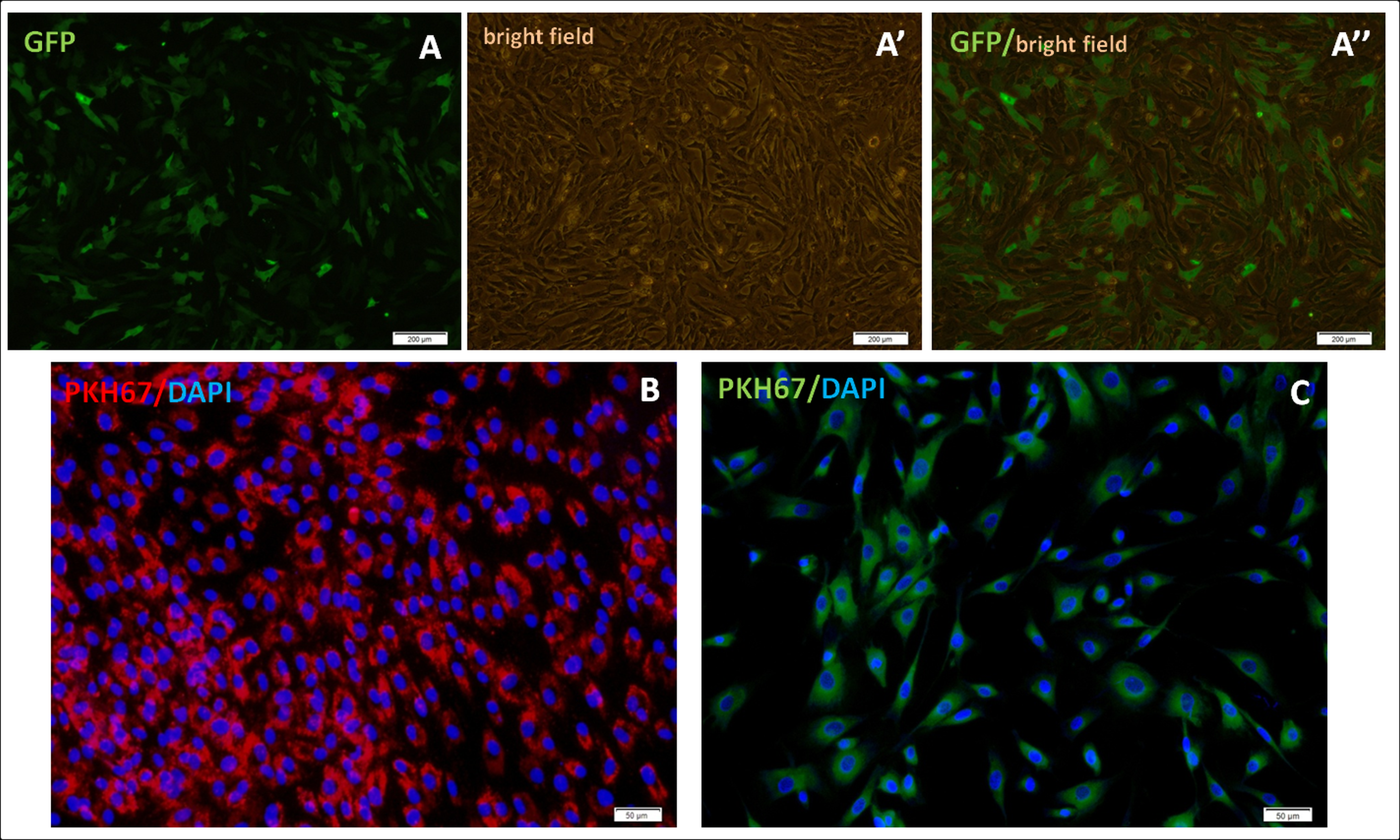

Supplement: S1 Fig — A, A', A'' represent the same field of view—caprine MSC two days after nucleofection with GFP encoding gene. Less than 50% of cells display distinct green fluorescence. B) Caprine MDC 2 days after labeling with PKH26 (100% efficiency); C) Caprine MSC 2 days after labeling with PKH67 (100% efficiency). Scale bars: A—200 μm, B,C—50 μm. (TIF) [file pone.0161693.s001.tif]

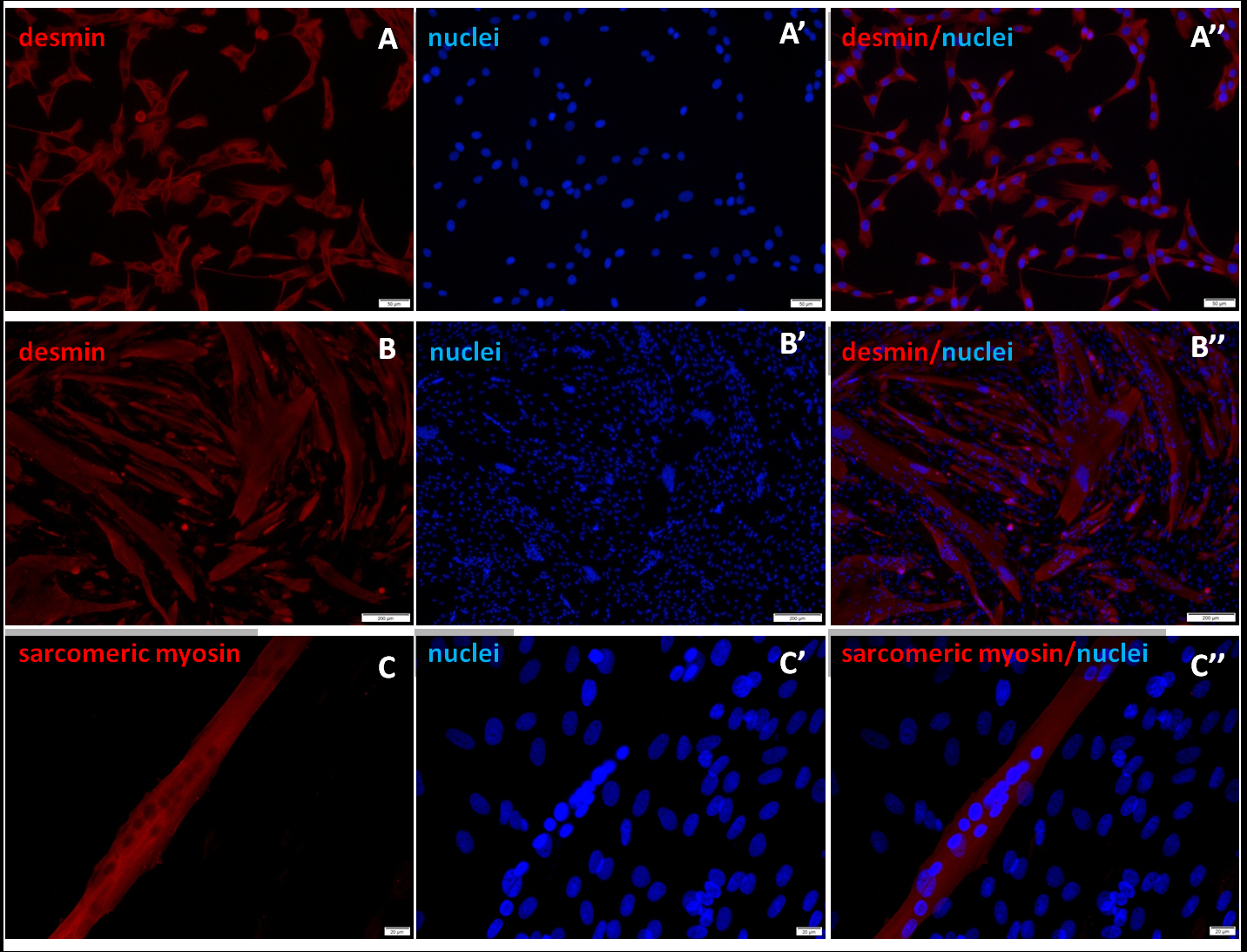

Supplement: S2 Fig — Images in rows represent the same field of view: A) Undifferentiated MDC; B, C) MDC differentiated into myotubes. A, B) Desmin is stained in red (Alexa Fluor® 594), nuclei are stained in blue (DAPI); C) Sarcomeric myosin is stained in red (Alexa Fluor® 594), nuclei are stained in blue (DAPI). Scale bars: A—50 μm, B,C—200 μm, C—20 μm. (TIF) [file pone.0161693.s002.tif]

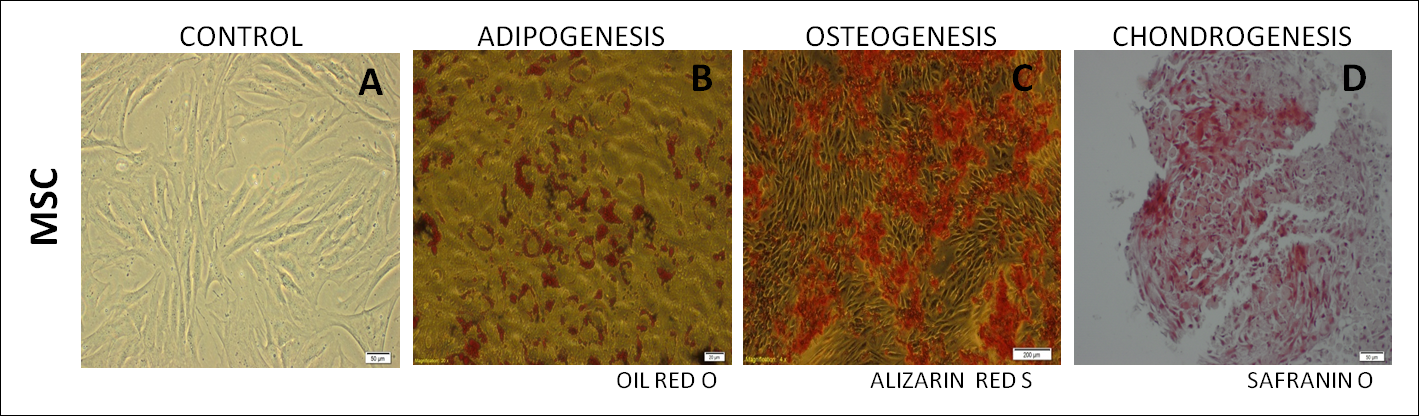

Supplement: S3 Fig — A) Undifferentiated MSC; MSC differentiated into adipocytes (B), osteocytes (C) and chondrocytes (D). (TIF) [file pone.0161693.s003.tif]
